# A meta-analysis of the clinical efficacy of Tanreqing injection combined with antibiotics vs antibiotics alone for treating pulmonary infection secondary to intracerebral hemorrhage

**DOI:** 10.1097/MD.0000000000024905

**Published:** 2021-03-19

**Authors:** Dongrui Zhou, Liandi Xie, Xiaowei Shi, Fengzhi Liu, Shuang Wu, Shuangshuang Zhang, Ruijia Liu, Jingling Chang, Lingqun Zhu

**Affiliations:** a Key Laboratory of Chinese Internal Medicine of Educational Ministry and Beijing, Dongzhimen Hospital; b Department of Cardiology, Dongfang Hospital, Beijing University of Chinese Medicine; c Department of Massage, The Third Affiliated Hospital of Beijing University of Chinese Medicine; d Department of Oncology, Beijing Daxing District Hospital of Integrated Chinese and Western Medicine; e Department of Neurology, Dongzhimen Hospital, Beijing University of Chinese Medicine, Beijing, China.

**Keywords:** intracerebral hemorrhage, meta-analysis, pulmonary infection, Tanreqing injection

## Abstract

**Background:**

Pulmonary infection is the most common complication to develop after intracerebral hemorrhage (ICH). Antibiotics have certain limitations when used to treat pulmonary infection, while Tanreqing injection (TRQI) is extensively used to treat pulmonary infection as an adjuvant to antibiotics. The aim of this meta-analysis was to investigate the clinical efficacy of TRQI for the treatment of lung infection secondary to ICH.

**Methods:**

Randomized controlled trials (RCTs) assessing the combination of TRQI and antibiotics compared to antibiotics alone for pulmonary infection after ICH were comprehensively searched for in 7 electronic databases from their establishment to August 2020. Two independent researchers conducted the literature retrieval, screening, and data extraction. The assessment tool of Cochrane risk of bias and Review Manager 5.3 software were applied to assess the methodological quality and analyze the data, respectively.

**Results:**

Seventeen RCTs involving 1122 patients with pulmonary infection after ICH were included. Compared to antibiotics alone, the combination treatment enhanced the clinical effective rate, shortened the hospital stay, reduced the white blood cell, procalcitonin, and C-reactive protein levels, ameliorated the times to the resolution of fever, cough, and lung rales, and increased the oxygenation index. The evidence indicated that TRQI combined with antibiotics caused no adverse reactions.

**Conclusions:**

Our study showed that the combination of TRQI and antibiotics was effective for treating pulmonary infection after ICH. However, high-quality multicenter RCTs are needed to further verify the clinical efficacy of TRQI due to the publication bias and the low methodological quality of the included RCTs.

## 1 Introduction

Intracerebral hemorrhage (ICH) refers to primary nontraumatic cerebral parenchymal hemorrhage, also known as spontaneous cerebral hemorrhage. ICH is a common subtype of stroke, accounting for 10% to 20% of all strokes, and it is characterized by high disability and mortality rates.^[1]^ In China, the incidence of ICH is 17% to 51%, far higher than in other countries (6%–20%).^[2]^ The mortality rate within 1 month of ICH is as high as 46.5%.^[3]^ Most surviving ICH patients have significantly limited function regarding daily activities, with only 12% to 39% regaining functional independence.^[4]^ In addition to the brain tissue damage caused by primary and secondary injuries, severe complications can also lead to poor function; the most common and most serious complication is lung infection.^[5]^

Many factors influence pulmonary infection after ICH. One type of factor is the patient's general condition and related factors, including the patient's gender,^[6]^ age, disturbances of consciousness, swallowing dysfunction, bed rest, vomiting, history of smoking, cardiac insufficiency, underlying lung diseases, and diabetes,^[7–9]^ The other type of factor relates to the use of preventive measures after hospital admittance, including nasal feeding therapy, ventilator therapy, and drug therapy.^[10–12]^ Once a patient with ICH develops pulmonary infection, the treatment duration and the hospital stay may be prolonged, the cost may increase, and the patient's life may even be endangered.^[13]^ Therefore, early diagnosis and timely treatment of lung infection after ICH significantly improves the prognosis of patients. However, the extensive use of antibiotics to prevent and treat pulmonary infection can very easily lead to drug resistance, and the treatment effects will decline.^[14,15]^

Traditional Chinese medicine preparations, with clear curative effects and high bioavailability,^[16,17]^ are widely used. Many studies have shown that combining Chinese medicine preparations and western medicines can produce synergistic effects.^[18–20]^ Tanreqing injection (TRQI) is a famous and effective Chinese medicine preparation. It is composed of *Scutellaria baicalensis, cornu saigae tataricae, fel ursi pulvis, flos lonicerae,* and *fructus forsythia*. It has antiviral, antibacterial, and anti-inflammatory effects,^[21]^ and can effectively treat pneumonia, upper respiratory tract infection, and chronic obstructive pulmonary disease.^[22–24]^ Experimental research has shown that TRQI not only has an antibacterial effect but it also synergistically increases the antibacterial activity of antibiotics when used in combination.^[25]^ Studies have shown that TRQI can effectively treat pulmonary infection that is complicated by other diseases.^[26,27]^ Although many clinical studies have shown that TRQI can be used to treat pulmonary infection secondary to ICH, its efficacy has not been systematically reviewed in a meta-analysis. Hence, we conducted a meta-analysis to assess the clinical curative effect of TRQI in patients with pulmonary infection secondary to ICH.

## 2 Methods

This meta-analysis was conducted in accordance with the Preferred Reporting Items for Systematic Reviews and Meta-Analyses (PRISMA)^[28]^ checklist.

### 2.1 Ethics

Since this study is a meta-analysis with no patient recruitment and personal information collection, the approval of the ethics committee is not required.

### 2.2 Inclusion criteria

Randomized controlled trials (RCTs) that met the following conditions were included:

1. Patients were diagnosed with pulmonary infection after ICH (with clear diagnostic criteria).
2. All patients received conventional antibiotics, such as ceftriaxone sodium, cefuroxime sodium, levofloxacin lactate, cefoperazone sodium, or sulbactam sodium. The control group was given antibiotics only, while the experimental group was given TRQI combined with antibiotics.
3. Outcomes included at least one of the following: the clinical effective rate (the primary outcome), length of hospital stay, procalcitonin (PCT), C-reactive protein (CRP), and white blood cell (WBC) levels, times to the resolution of fever, cough, and lung rales, oxygenation index (OI), and adverse reactions (ADRs). The clinical effective rate was calculated using the following formula: (number of cured patients + number of patients with notable improvement + number of patients with improvement) / total number of patients × 100%.
4. The study was an RCT.

### 2.3 Exclusion criteria

The exclusion criteria included:

1. Non-RCTs.
2. The patients had other cerebrovascular diseases complicated with pulmonary infection.
3. Incomplete or incorrect data.
4. The outcome data could not be analyzed.

### 2.4 Search strategy

Two independent researchers conducted literature searches using the China Science and Technology Journal Database (VIP), Chinese Biomedical Literature Database (CBM), Wanfang Database, China National Knowledge Infrastructure (CNKI), Cochrane Library, PubMed, and Embase from database establishment to August 2020, with no language restrictions. The search terms were as follows: (#1) cerebral hemorrhage OR hematencephalon OR encephalorrhagia OR intracerebral hemorrhage OR brain hemorrhage OR ICH; (#2) pulmonary infection OR pneumonia OR lung infection; (#3) Tanreqing OR Tanreqing injection OR TRQI; (#4) #1 AND #2 AND #3. The references of the included articles were also searched to avoid missing any relevant RCTs.

### 2.5 Screening, data extraction, and risk of bias assessment

The retrieved articles were screened for potential inclusion by 2 independent researchers (XWS and SW), and if any disagreements were encountered, a third researcher (LDX) resolved them. Regarding the included RCTs, the extracted data were as follows:

1. first author and year of publication;
2. sample size, gender distribution, and mean age;
3. types, dosages, and duration of treatments;
4. outcomes and ADRs.

### 2.6 Risk of bias assessment

The Cochrane risk of bias tool was used to assess the methodological quality of the included RCTs.^[29]^ The tool includes 7 items:

1. generation of random sequence;
2. concealment of the allocation plan;
3. blinding of subjects and researchers;
4. blinding of outcome evaluators;
5. completeness of the outcome data;
6. selective reporting of the results;
7. other sources of bias.

Each item is evaluated as unclear, high risk, or low risk. If any disagreements were encountered, a third researcher (LDX) resolved them.

### 2.7 Statistical analysis

Review Manager 5.3 software was used to analyze the data. Dichotomous variables are presented as odds ratios (ORs) and continuous variables as mean differences (MDs). Additionally, 95% confidence intervals (CIs) were calculated for these statistics. Qualitative and *I*^2^ statistics were used to evaluate the heterogeneity among the RCTs. If the heterogeneity across studies was low (*P* ≥ .1 and *I*^2^ < 50%), a fixed-effect model was applied for data analysis. If the heterogeneity across studies was high (*P* < .1 and *I*^2^ > 50%), a random-effects model was applied to analyze the data. The sources of heterogeneity were confirmed using subgroup analysis. Publication bias was planned to be assessed using a funnel plot if there were >10 studies.

## 3 Results

### 3.1 Search results

**Figure 1:**
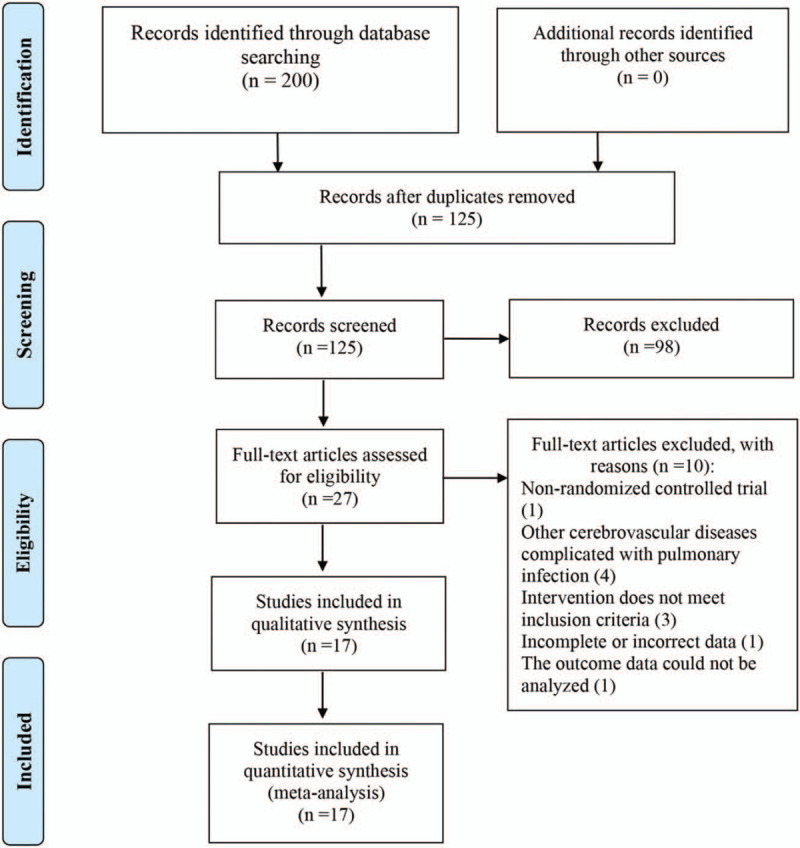
Literature retrieval and screening process.

Based on the search strategy, we retrieved a total of 200 articles after searching 7 databases, comprising 119 from Wangfang, 37 from CBM, 25 from CNKI, and 19 from VIP (no relevant literature was retrieved from PubMed, Cochrane Library, or Embase). After eliminating duplicates and screening the titles and abstracts, 27 documents were obtained for further screening. There were 10 documents that did not meet the inclusion criteria; the details are as follows: not RCTs (1), the patients had other cerebrovascular diseases complicated with pulmonary infection (4), the intervention did not meet the inclusion criteria (3), incomplete or incorrect data (1), and the outcome data could not be analyzed (1). Finally, 17 RCTs were enrolled in the meta-analysis. Figure 1 shows the literature retrieval and screening process.

### 3.2 Study characteristics

A total of 17 RCTs involving 1122 patients were included, all of whom were from mainland China, comprising 565 in the experimental groups and 557 in the control groups. The maximum and minimum sample sizes were 100 and 30, respectively. The dosage of TRQI was 20 ml once per day (10 RCTs) or 30 ml once per day (7 RCTs). The treatment duration ranged from 7 to 16 days. TRQI was administered by intravenous drip in all cases. Table 1 shows the characteristics of each RCT.

**Table 1:**
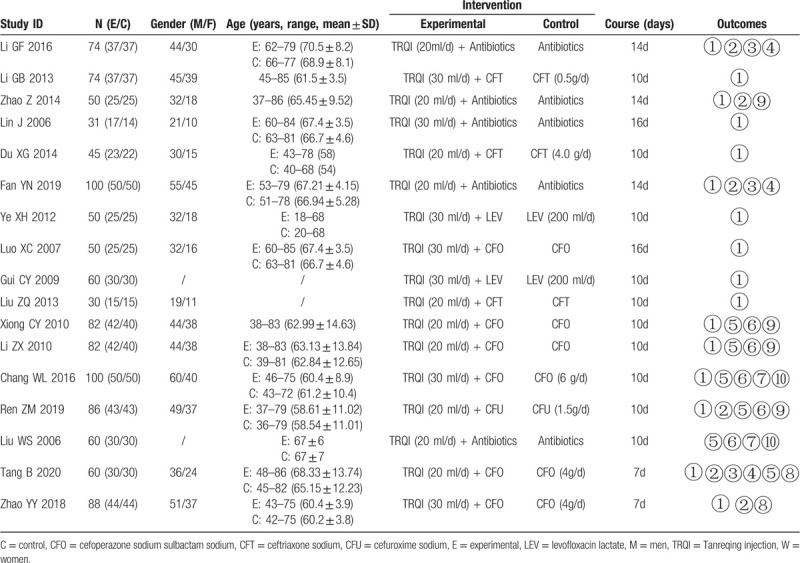
Characteristics of the included RCTs.

### 3.3 Quality assessment of included RCTs

**Figure 2:**
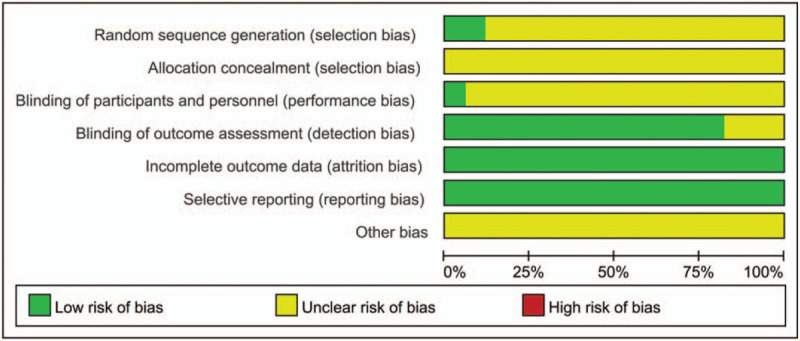
Risk of bias of enrolled RCTs.

**Figure 3:**
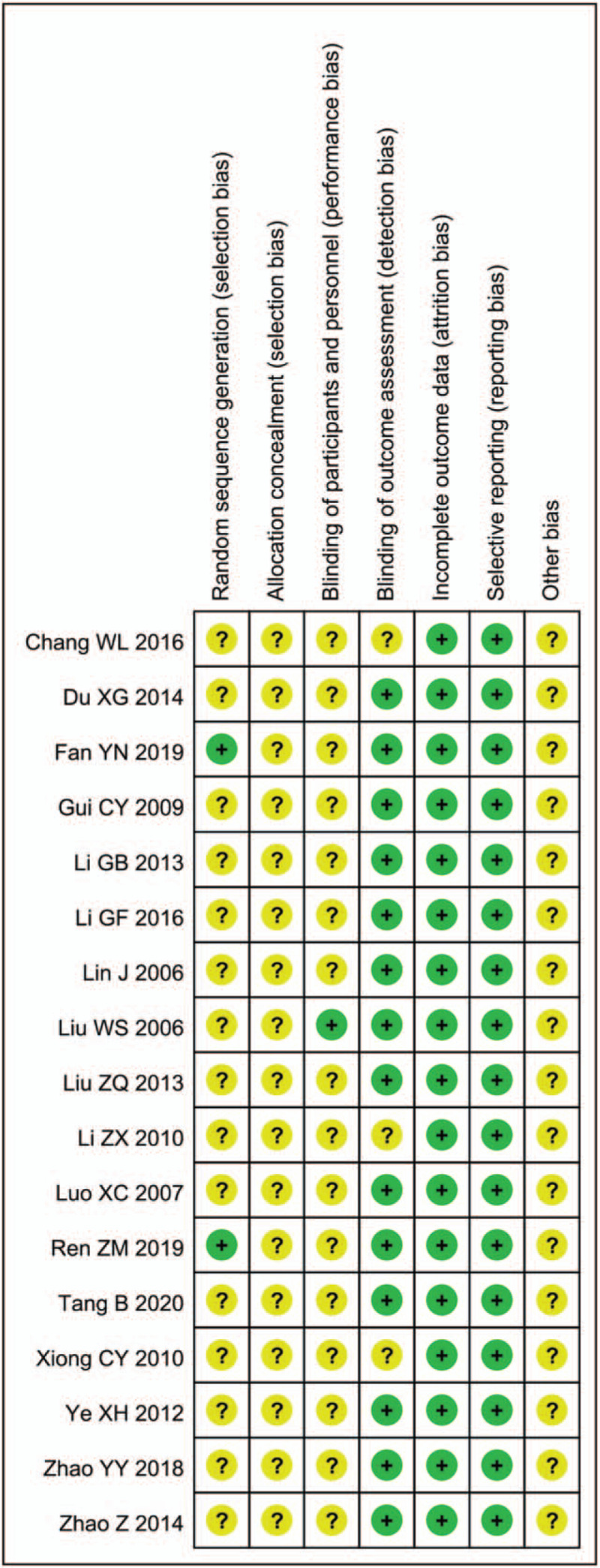
Summary of risk of bias.

Two studies (which were considered low risk) clearly illustrated the method of random sequence generation; the remaining 15 studies (which were considered of unclear risk) described the method used to generate random sequences as “random” but did not describe the specific randomization method. All of the studies (which were considered of unclear risk) failed to mention allocation concealment. One study (which was considered low risk) blinded the subjects and researchers; the remaining studies (which were considered of unclear risk) did not mention blinding of the subjects and researchers. All of the studies (which were considered of unclear risk) failed to clarify whether there were other biases. Figures 2 and 3 show the risk of bias of the enrolled RCTs.

### 3.4 Outcomes of the meta-analysis

#### 3.4.1 Clinical effective rate

**Figure 4:**
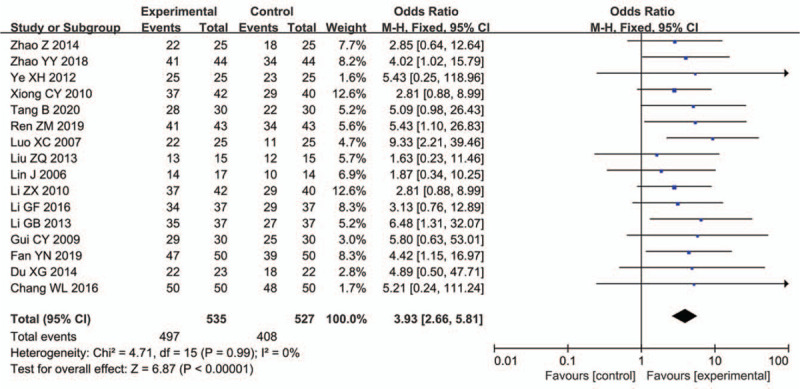
Comparison of clinical efficacy.

Sixteen studies^[30–45]^ involving 1062 patients reported the clinical effective rate of pulmonary infection secondary to ICH. The results of the meta-analysis showed that the clinical effective rate of TRQI combined with antibiotics was superior to that of antibiotics alone (OR: 3.93, 95% CI: 2.66, 5.81; *P* < .00001) (Fig. 4).

#### 3.4.2 Length of hospital stay

**Figure 5:**
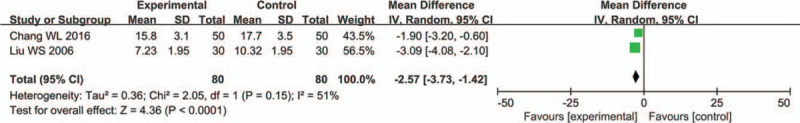
Comparison of length of hospital stay.

Two RCTs^[30,46]^ involving 160 patients reported the length of hospital stay. Based on the obvious heterogeneity among the included RCTs (*P* = .15, *I*^2^ = 51%), a random-effects model was applied for analysis. The results displayed that TRQI adjuvant treatment effectively reduced the length of hospital stay (MD: −2.57, 95% CI: −3.73, −1.42) (Fig. 5).

#### 3.4.3 CRP level

**Figure 6:**
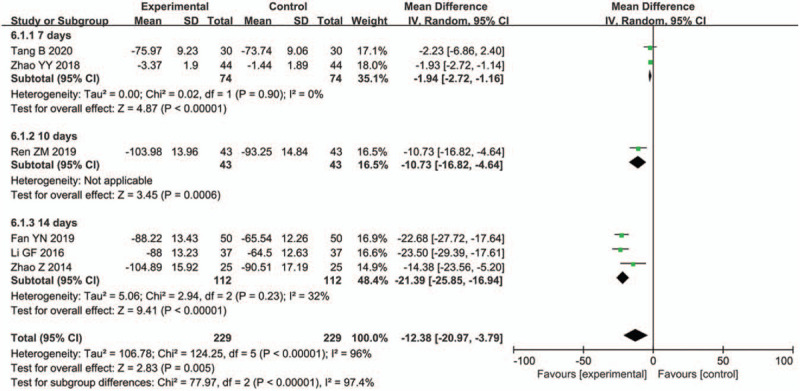
Comparison of C-reactive protein (CRP) level.

Six studies^[32,35,40,41,44,45]^ involving 458 patients assessed the CRP level in patients with pulmonary infection after ICH. As there was obvious heterogeneity, a subgroup analysis was conducted according to treatment duration. TRQI combined with antibiotics was found to clearly decrease the CRP level at 7 days (MD: −1.94, 95% CI: −2.72, −1.16; *P* < .00001), 10 days (MD: −10.73, 95% CI: −16.82, -4.64; *P* = .0006), and 14 days (MD: −21.39, 95% CI: −25.85, −16.94; *P* < .00001). Thus, the results indicate that the heterogeneity may be related to the varying treatment durations (Fig. 6).

#### 3.4.4 WBC and PCT levels

**Figure 7:**
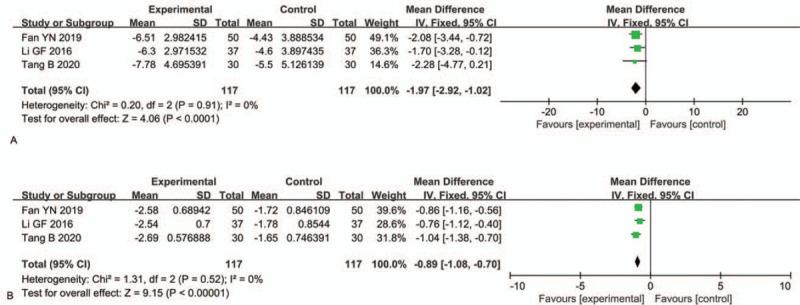
Comparisons of (A) white blood cell (WBC) and (B) procalcitonin (PCT) levels.

WBC and PCT levels were assessed in 3 RCTs^[32,35,41]^ involving 234 patients. The combination of TRQI and antibiotics effectively reduced the WBC and PCT levels compared to antibiotics alone (MD: −1.97, 95% CI: −2.92, −1.02, *P* < .0001; MD: −0.89, 95% CI: −1.08, −0.70, *P* < .00001) (Fig. 7).

#### 3.4.5 Time to resolution of fever

**Figure 8:**
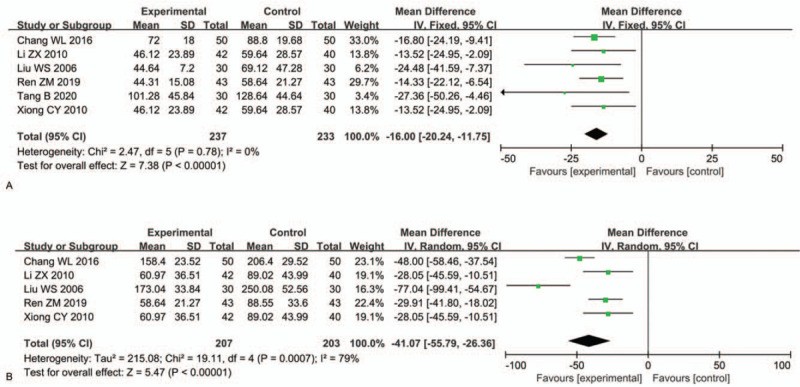
Comparisons of the time to resolution of (A) fever and (B) cough.

Six RCTs^[30,36,40–42,46]^ involving 470 patients assessed the time to the resolution of fever. The time to the resolution of fever in the experimental group was shorter than that in the control group (MD: −16.00, 95% CI: −20.24, −11.75; *P* < .00001) (Fig. 8A).

#### 3.4.6 Time to resolution of cough

Five RCTs^[30,36,40,42,46]^ involving 410 patients reported the time to the resolution of cough. Due to the obvious heterogeneity (*P* = .0007, *I*^2^ = 79%), a random-effects model was used for data analysis. TRQI combined with antibiotics relieved cough more rapidly than antibiotics alone (MD: −41.07, 95% CI: −55.79, −26.36; *P* < .00001) (Fig. 8B).

#### 3.4.7 Time to resolution of lung rales

**Figure 9:**
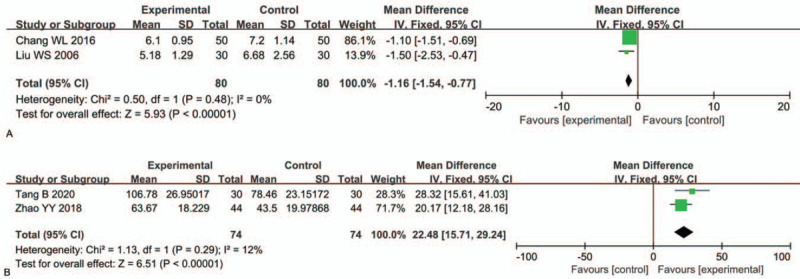
Comparisons of (A) time to resolution of lung rales and (B) oxygenation index (OI).

Two RCTs^[30,46]^ involving 160 patients reported the time to the resolution of lung rales. TRQI combined with antibiotics had obvious an advantage regarding shortening the time to the resolution of lung rales compared to antibiotics alone (MD: −1.16, 95% CI: −1.54, −0.77; *P* < .00001) (Fig. 9A).

#### 3.4.8 Oxygenation index

Two RCTs^[41,44]^ involving 148 patients reported the OI. TRQI combined with antibiotics significantly increased the OI compared to antibiotics alone (MD: 22.48, 95% CI: 15.71, 29.24; *P* < .00001) (Fig. 9B). This result suggested that TRQI adjuvant therapy significantly increased the OI of patients with lung infection after ICH.

#### 3.4.9 ADRs

Of all the 17 RCTs included in this study, 4 reported that there were no ADRs and the remaining 13 did not report the ADRs.

### 3.5 Publication bias

**Figure 10:**
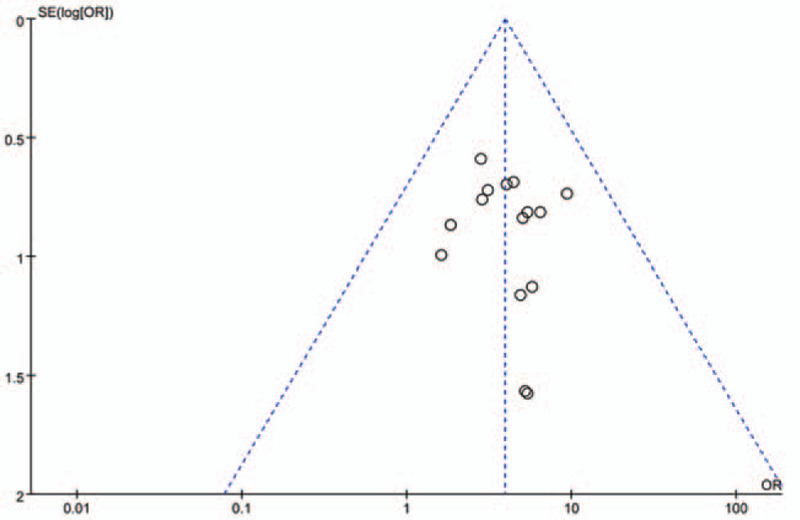
Funnel plot of clinical efficacy.

Based on clinical effective rate, a funnel plot was established to evaluate publication bias. Figure 10 indicates evidence of a small degree of publication bias.

## 4 Discussion

This study of 17 RCTs, involving 1212 patients, provides evidence regarding the efficacy of the combination of TRQI and antibiotics for the treatment of pulmonary infection secondary to ICH. We found that TRQI combined with antibiotics may be a safe and effective treatment for pulmonary infection after ICH, as it improved the clinical curative effect, shortened the patients’ hospital stay, decreased inflammation levels, and ameliorated other clinical symptoms and signs, and no ADRs were reported.

ICH is an acute and serious diseases, which is characterized by rapid onset, rapid changes in the patient's condition, and high disability and fatality rates.^[47]^ A case of ICH can easily become complicated with pulmonary infection during treatment, which impacts the treatment effects, prolongs the hospital stay, and can even lead to the death of the patient.^[48]^ Therefore, prevention and appropriate anti-infective treatment are critical to the patient prognosis. In this meta-analysis, we investigated the clinical efficacy of the combination of TRQI and antibiotics in patients with pulmonary infection secondary to ICH. The results showed that the clinical curative effect of the combined treatment was obviously superior to the effect of antibiotics alone, and the hospital stay was also evidently shortened. Long-term use of antibiotics can lead to drug resistance, reducing the effectiveness of the antibiotics. However, TRQI can be used in combination with antibiotics in the treatment of pulmonary infection secondary to ICH, shortening the treatment time and increasing the clinical efficacy.

CRP, WBC, and PCT levels are sensitive indicators of lung infection, being increased during the process of lung infection, and they are often used to monitor patients’ conditions.^[49,50]^ Our results showed that TRQI combined with antibiotics clearly reduced the CRP, WBC, and PCT levels compared to antibiotics alone, which indicates that TRQI has anti-inflammatory effects.

Fever is the most persistent and usually the only indicator of pulmonary infection,^[51]^ while cough, sputum, and lung rales are common symptoms and signs of pneumonia.^[52]^ The OI is an indicator that prompts the patient's pulmonary ventilation function, and it can reflect hypoxia and lung injury.^[53]^ Our study showed that after treatment with TRQI combined with antibiotics, the times to the resolution of fever, cough, and lung rales were shorter than those associated with the use of antibiotics alone. Additionally, the combination treatment of TRQI and antibiotics could evidently increase the OI compared to the antibiotics alone. This suggested that TRQI combined with antibiotics had advantages over antibiotics alone regarding alleviating the clinical symptoms and signs in patients with pulmonary infection secondary to ICH. Chlorogenic acid, baicalin, ursodeoxycholic acid, and chenodeoxycholic acid are the main active ingredients in TRQI, and they have obvious antibacterial and anti-inflammatory effects.^[54,55]^ Studies have shown that TRQI not only has significant effects on community-acquired pneumonia, chronic obstructive pulmonary disease, and bronchitis,^[56]^ but it also has excellent curative effects on pneumonia after lung cancer,^[26]^ pulmonary tuberculosis,^[27]^ and stroke.^[57]^

ADRs must be considered in addition to the clinical benefits when assessing the clinical effects of a medication. This study showed that TRQI combined with antibiotics appears to be a safe treatment. However, only 4 of the 17 (23.53%) RCTs reported on ADRs (all 4 reported that there were no ADRs), so definitive conclusions cannot be drawn. The ADRs listed in the TRQI instructions mainly include dizziness, nausea, vomiting, allergic reactions, and itching or rash, which are mainly related to the age and physique of the patients, solvent used, medications that TRQI is combined with, and intravenous drip speed.^[58]^ Therefore, during the administration of TRQI, medical staff should pay close attention to the clinical reactions of the patients and instruct them to be aware of possible ADRs after taking TRQI. If ADRs appear, TRQI should be immediately discontinued and treatment should be provided for the ADRs, if necessary.

The overall methodological quality of the RCTs enrolled in this study was not high. All of the studies were randomized, but no specific randomization method was described for 15 of them. None of the RCTs mentioned allocation concealment and none clarified whether there were other biases. Only 1 RCT reported double-blinding (of subjects and researchers). These issues may weaken the reliability of the evidence in our study.

Based on clinical efficacy, we created a funnel plot to assess publication bias and found that it was asymmetric, indicating a small degree of publication bias. Although we conducted a comprehensive literature search, this study lacks the support of other languages, unpublished studies and grey articles, which may lead to selection bias.

This meta-analysis objectively assessed the clinical efficacy of TRQI in combination with antibiotics, and it provides medical evidence for the therapeutic use of TRQI for pulmonary infection secondary to ICH. However, there were several limitations. Since TRQI is exclusively used in China, therefore, the recruited patients in the RCTs were all from China, with a lack of patients from other countries. Some of the RCTs had small sample sizes, so it is uncertain whether they had any influence on the assessment of the clinical efficacy of the combined treatment of TRQI and antibiotics. Hence, high-quality multicenter RCTs are needed to provide more solid evidence for the use of TRQI.

## 5 Conclusions

This meta-analysis showed that the combination of TRQI and antibiotics may improve clinical efficacy, shorten the hospital stay, reduce inflammation levels, and ameliorate other clinical symptoms and signs, and the evidence indicated that the combination treatment is safe. However, due to the small sample sizes, low methodological quality, and potential publication bias related to the included RCTs in this study, large high-quality multicenter RCTs are needed to further verify the efficacy of TRQI for treating lung infections after ICH.

## Author contributions

**Conceptualization:** Dongrui Zhou, Lingqun Zhu.

**Data curation:** Ruijia Liu.

**Formal analysis:** Fengzhi Liu, Shuangshuang Zhang.

**Funding acquisition:** Jingling Chang.

**Methodology:** Xiaowei Shi, Fengzhi Liu, Shuang Wu.

**Software:** Dongrui Zhou.

**Supervision:** Liandi Xie, Lingqun Zhu.

**Visualization:** Liandi Xie.

**Writing – original draft:** Dongrui Zhou.

**Writing – review & editing:** Liandi Xie, Xiaowei Shi, Jingling Chang.
